# Emerging Role of Mucosal Vaccine in Preventing Infection with Avian Influenza A Viruses

**DOI:** 10.3390/v12080862

**Published:** 2020-08-07

**Authors:** Tong Wang, Fanhua Wei, Jinhua Liu

**Affiliations:** 1 Key Laboratory of Animal Epidemiology and Zoonosis, Ministry of Agriculture, College of Veterinary Medicine, China Agricultural University, Beijing 100193, China; wtong0819@163.com; 2 College of Agriculture, Ningxia University, Yinchuan 750021, China; weifanhua999@163.com

**Keywords:** influenza virus, poultry, upper respiratory tract, mucosal immunity, mucosal vaccines

## Abstract

Avian influenza A viruses (AIVs), as a zoonotic agent, dramatically impacts public health and the poultry industry. Although low pathogenic avian influenza virus (LPAIV) incidence and mortality are relatively low, the infected hosts can act as a virus carrier and provide a resource pool for reassortant influenza viruses. At present, vaccination is the most effective way to eradicate AIVs from commercial poultry. The inactivated vaccines can only stimulate humoral immunity, rather than cellular and mucosal immune responses, while failing to effectively inhibit the replication and spread of AIVs in the flock. In recent years, significant progresses have been made in the understanding of the mechanisms underlying the vaccine antigen activities at the mucosal surfaces and the development of safe and efficacious mucosal vaccines that mimic the natural infection route and cut off the AIVs infection route. Here, we discussed the current status and advancement on mucosal immunity, the means of establishing mucosal immunity, and finally a perspective for design of AIVs mucosal vaccines. Hopefully, this review will help to not only understand and predict AIVs infection characteristics in birds but also extrapolate them for distinction or applicability in mammals, including humans.

## 1. Introduction

Avian influenza A viruses (AIVs) remain one of the important viral pathogens that cause respiratory tract infections in various animal species, such as poultry and humans, and aquatic birds are considered the primordial source of AIVs infecting other animal species [1]. Influenza A virus (IAV) is a single-stranded negative-sense RNA virus of the family Orthomyxoviridae that is grouped into the following four genera: A–D. IAV is classified into distinct subtypes based on its two surface glycoproteins: hemagglutinin (HA) (18 subtypes) and neuraminidase (NA) (11 subtypes) [2,3,4]. AIVs are categorized as either highly pathogenic or low pathogenic viruses based on the presence of a polybasic cleavage site in the HA protein as well as the disease severity in poultry. Infection with low pathogenic avian influenza virus (LPAIV) results in mild respiratory disorder accompanied with reduced egg production and/or a depression, whereas infections with highly pathogenic avian influenza virus (HPAIV) cause a significant mortality in chickens. Notably, AIVs, particularly subtypes H5, H7 and H9, have posed an increasing threat to public health worldwide. So far, H5N1 and H7N9 have caused hundreds of human infections, with a mortality rate of 20% to 60% [5,6]. Importantly, the H7N9 virus remains a potential pandemic threat [7]. Meanwhile, H9N2 has been identified as the most popular AIVs strain that can cause heavy economic losses and mutate into highly pathogenic forms as well [8].

Influenza viruses undergo antigen mutations through antigen drift and shift. Circulating AIVs evolve constantly, resulting in the occurrence of new strains with variable antigenicity. Particularly, mutations occurring in the HA or NA usually cause antigenic drift, subsequently leading to an escape from earlier immune responses. Besides, an antigenic shift can take place in AIVs. In this case, co-infection of a host cell with AIVs strains of different origins can lead to the generation of novel virus subtypes via induced genetic reassortment; a newly formed HA subtype of AIVs can transmit easily in immunologically naïve individuals [9]. To date, there are very few cases of human–human transmission of AIVs. Overall, AIVs still have the potential to cause a pandemic, thereby presenting one of the greatest challenges in public health [10,11,12].

Vaccination remains one of the most effective ways to control and prevent AIVs transmission in poultry. Current influenza vaccines are generally effective against antigenically matched strains. It has been demonstrated that the antigenic mismatch between the vaccine and circulating strains or inadequate vaccine protocols can cause an antigenic drift and failed vaccination [13,14]. Although the strategy of inactivated vaccine immunization has been conducted for more than 10 years, AIVs are still prevalent in poultry. It has been proposed that extensive vaccination for the control of AIV epidemics in poultry favors the occurrence of antigenically diverse viruses drifted from the seasonal vaccine strains, possibly reducing vaccine efficacy and causing failed vaccination [15,16]. Compared to inactive vaccines, mucosal vaccines evoke a better local immune response [17]. Given that the infection and spread of AIVs occur at mucosal surfaces, vaccine administration via the mucosal route could provide the first line of defense against viral infections through inducing mucosal immunity. Moreover, mucosal vaccines are designed to target specific mucosal tissues and to trigger “frontline immunity” for IgA production in the upper respiratory tract (URT), controlling viral infection. Therefore, understanding the underlying mechanisms for mucosal immunity would contribute to design and adopt more adequate vaccine strategies for the control of AIVs infection and epidemics.

## 2. Host Immune Responses to Influenza Viruses

### 2.1. Innate Immune Response to Influenza Virus

**Figure 1:**
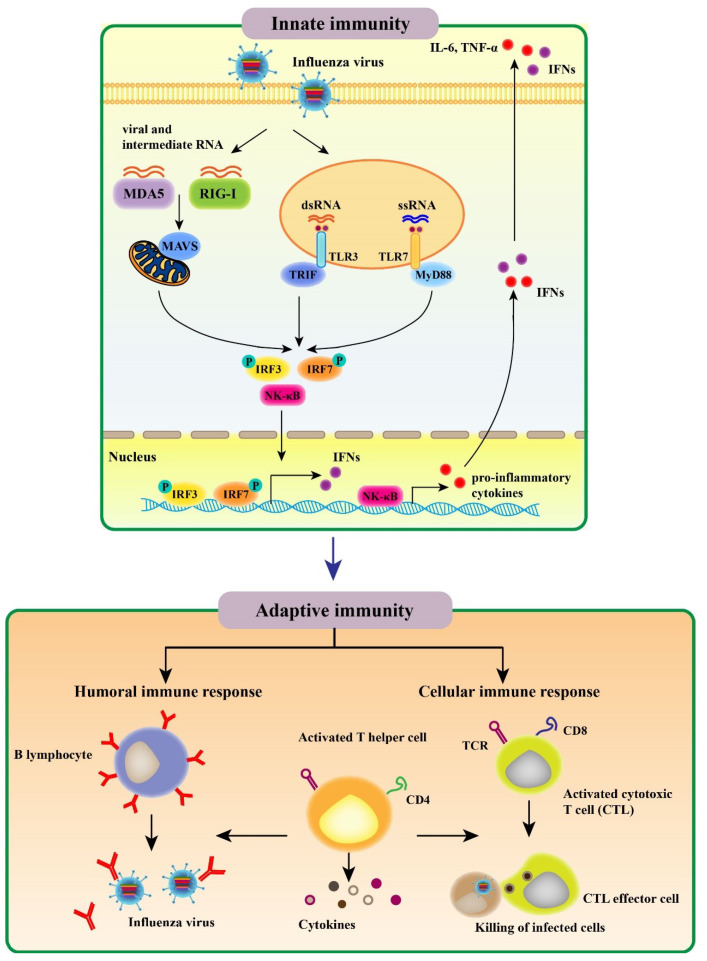
The innate and adaptive immunity against influenza virus.

The innate immune response constitutes the first line of protection from invading pathogens. AIV infection results in a recognition of viral conserved components called pathogen associated molecular patterns (PAMPs) by host pathogen recognition receptors (PRRs), including Toll-like receptors (TLRs), retinoic acid inducible gene-I (RIG-I) receptors (RLRs), and NOD-like receptors (NLRs); the above recognition subsequently activates innate immune signaling and induces various proinflammatory cytokines as well as antiviral molecules [18,19]. Then, activation of the initial innate immune response regulates the adaptive immune system (Figure 1).

TLRs were identified as the first PRRs family that are expressed on either the plasma membrane (TLRs 1–6, 10, and 11) or the surface of endosomes and lysosomes (TLR3, 7, 8, and 9) [20]. To date, 10 human, 13 murine, and 10 chicken TLRs have been identified and characterized. In chicken, eight TLRs have been shown to recognize a range of molecules, including diacylated lipopeptides (TLR1), triacylated lipopeptides (TLR2), double stranded RNA (dsRNA) (TLR3), LPS (TLR4), flagellin (TLR5), single stranded RNA (ssRNA) (TLR7), yeast proteases (TLR15), and double stranded DNA (dsDNA) (TLR21) [21]. For influenza virus infection, endosomal TLR3 and TLR7 recognize viral infection-produced dsRNA and ssRNA genome, respectively (Figure 1). TLR3 is localized on the surface of bronchial and alveolar epithelial cells as well as to their endosomes. TLR3 pathway activates the transcription factors, including nuclear factor kappa-B (NF-κB) and interferon regulatory factor 3 (IRF3), inducing the expression of type I interferons (IFNs) and proinflammatory cytokines. TLR7 is a receptor for influenza viral ssRNA in endolysosomal compartments of plasmacytoid dendritic cells (pDCs). An adaptor protein MyD88 mediates TLR7 signaling transduction, activating IRF7 and NF-κB [22] (Figure 1). Overall, cytokine production promotes the expression of major histocompability complex (MHC) and co-stimulatory molecules in antigen-presenting cells (APCs), which regulates T helper (Th) cell differentiation and facilitates the immune responses to antigens. These properties of TLR signaling make TLR ligands promising candidates for vaccine adjuvants.

The RLRs—RIG-I and melanoma differentiation-associated protein 5 (MDA5)—recognize the cytoplasmic viral non-self RNA and transcripts. RIG-I preferentially binds cytoplasmic single-strand 5′-triphosphate RNA, while MDA5 is activated by long dsRNA species [23]. Upon influenza virus infection, the repressor domain (RD) of RIG-I recognizes the viral RNA molecule, resulting in the release of RD-mediated autoinhibition of caspase activation and recruitment domains (CARDs) in RIG-I. After the CARDs are dephosphorylated or ubiquitinated by TRIM-containing protein 25 (TRIM25) known as an E3 ligase [24], RIG-I associates with an adaptor molecule called mitochondrial antiviral signaling protein (MAVS) [25]. Then, the MAVS elicits a signaling cascade that ultimately activates transcription factors such as IRF3 to induce type I IFNs expression [26,27]. Although RIG-I is absent in chickens, the absence of chick RIG-I can be functionally compensated by MDA5. In ducks and geese, RIG-I displays a high degree of structural similarity with that in mammals [28].

The roles of NLR signaling in bacterial infection have been broadly investigated [29]. IAV infection activates NLR family pyrin domain containing 3 (NLRP3) and NLR apoptosis inhibitory protein 5 [30]. Notably, viral PB1-F2 protein acts as the signal activating the NLRP3 inflammasome [31]. Moreover, the IAV M2 ion channel is needed to activate the NLRP3 inflammasome and cleave pro-IL-1β and pro-IL-18 [32]. Overall, the function of NLR signaling is undisputable and complex. In a recent study, Ichinohe et al. found that NLRP3 inflammasome signaling is involved in the protective adaptive responses to IAV infection [33]. Conversely, other studies demonstrated that the NLRP3 signaling has no effect on the adaptive immune response [34,35].

### 2.2. Adaptive Immune Response to Influenza Virus

The adaptive immune response comprising humoral and cellular responses at both systemic and mucosal levels constitutes the second line of defense against influenza viruses; this multifaceted immune response is complex (Figure 1) [36]. In the case of the humoral immune response, influenza virus infection leads to production of antibodies (Abs) against the viral surface glycoproteins, particularly HA. Abs raised against the globular domain of trimeric HA could prevent the binding of infected viruses to the cell receptors in the host and endocytosis mediated by the receptors. As most Abs recognizing the viral HA exhibit virus strain-specific activities, they are ineffective in neutralizing viruses of other subtypes and intrasubtypic drift variants [37]. NA has a crucial role in the release and transmission of newly formed virions due to the presence of sialydase activities. NA Abs inhibit the viral spread and decrease the disease severity through suppressing the enzymatic activities [38]. It should be noted that Abs against nucleoprotein (NP) facilitate protective immune response to influenza viruses in mice [39]. Besides, it has been reported that matrix protein 1 (M1)- and NP-specific Abs stimulate the activity of natural killer cells [40].

Infection with influenza viruses initiates virus-specific cellular immune response involving CD4+ T and CD8+ T cells (Figure 1). Upon infection, the migration of dendritic cells (DCs) from the lungs to the draining lymph nodes was induced, resulting in the activation of CD4+ T cells [41,42]. Then, activated CD4+ T cells could differentiate into a variety of Th cell subsets in response to distinct stimulatory factors produced by epithelial cells, DCs, and inflammatory cells [43]. Th1 cells secret cytokines (IFN-γ, TNF, and IL-2), which promote cytotoxic T lymphocyte (CTL) response [44] and play an essential role in inducing memory CD8+ T cells [45]. Meanwhile, Th2 cells produce cytokines (IL-4, IL-5 and IL-13) that facilitate the activation and differentiation of B cells leading to Ab generation [46]. Memory CD4+ T cells play a role in controlling the secondary influenza infection that is independent of CD8+ T cells or B cells while contributing to innate immune response [47]. Strikingly, lung-resident memory CD4+ T cells, a new defined population, was identified to facilitate optimal protective response to influenza viruses [48]. Moreover, CD4+ T cells can be differentiated into Th17, regulatory T (Treg) cells, follicular helper T (Tfh) cells, and even killer cells depending on the local stimulators. Th17, Treg, and Tfh cells have been found to play a regulatory role in cellular immune response to IAVs [49,50].

During IAV infection, CD8+ T cells, a vital subset of immune cells, are activated after recognition of viral epitopes on DCs that migrate from the lung tissue to T-cell areas in the draining lymph nodes, resulting in their proliferation and differentiation into CTLs [51]. CD8+ T cell differentiation toward CTLs can also be induced by proinflammatory cytokines, including IFN-γ, type I IFNs, IL-2, and IL-12 [52]. CTLs have the capacity to inhibit progeny virus formation and spread by migrating to the infected site and eliminating cells infected with influenza virus [53]. In this case, CTLs release cytotoxic granules containing perforin and granzymes such as GrA and GrB, inducing apoptosis of the virus-infected cells [54]. Moreover, GrA prevents virus replication and exerts non-cytotoxic effects through cleaving viral and host cell proteins related to protein synthesis [55]. Meanwhile, production of pro-inflammatory cytokines, such as TNF-α, suppresses virus replication and promotes lytic activities [54]. In addition, CTLs can recruit death receptors of cells infected with IAVs via cytokines, including FASL, TNF, and TRAIL, causing Fas/FasL interaction–mediated apoptosis in the infected cells [56]. Neutralizing Abs provide protection against reinfection with the same serotype of influenza virus, and CTLs are mainly directed against epitopes of the highly conserved internal viral proteins, like M1, NP, polymerase acid (PA), and polymerase basic 2 (PB2). Thus, CTL responses contribute to heterosubtypic immunity and display cross-reactivities among various subtypes of IAVs [57].

### 2.3. Mucosal Immune Response to Influenza Virus

**Figure 2:**
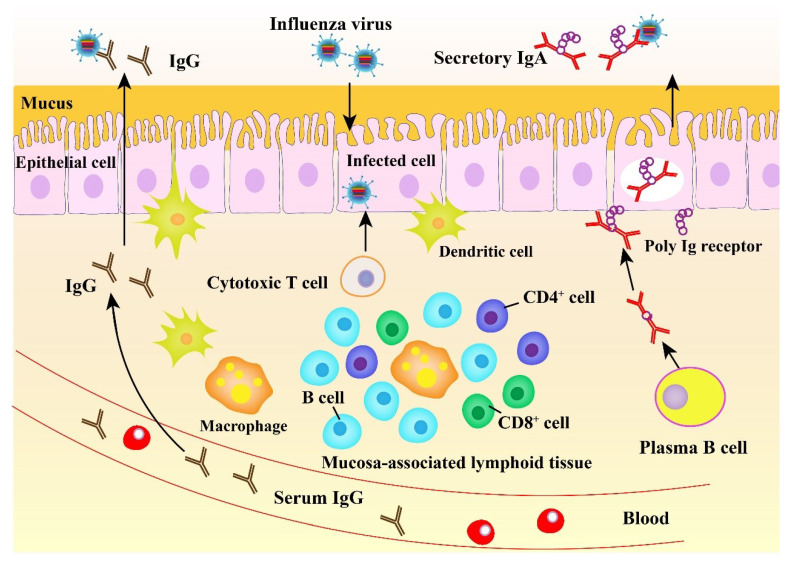
The mucosal immunity against influenza virus.

The mucosal immune system plays a crucial role in controlling infection with influenza viruses in the URT (Figure 2). The URT serves as the initial site for IAV entry and multiplication in the host and also plays a crucial location for host defense. A powerful oral-pharyngeal immune system in the host responds to the initial IAV infection by producing antiviral and immunomodulatory components, including mucins, lectins, antimicrobial peptides, complement molecules, and natural immunoglobulins [58]. The entry of IAVs into epithelial cells renders detection of the viral PAMPs by the local immune cells via PRRs. The above immune cells can induce antigen-specific immune responses, producing a variety of cytokines and chemokines acting in antiviral immunity and adaptive effector cell recruitment and activation.

Abs, particularly IgG and IgA, are the major immune components acting in the protective response to IAVs (Figure 2). IgG is secreted into blood and then diffuses onto the mucosal tissue. In the mucosa, IgG plays a major role in decreasing viral pneumonia rather than preventing the upper respiratory system infections [59]. In contrast to IgG, IgA exerts a key role in preventing from infection of the human URT mucosa with influenza viruses [60], eliminating viruses from the infected secretory epithelium, and relocating antigens from the lamina propria to the lumen [61]. Importantly, infected viruses can be neutralized by secretory IgA (S-IgA) on the surface of the respiratory mucosa prior to their attachment to epithelium and penetration into the epithelial surface (Figure 2). In addition, influenza-virus-induced S-IgA in the respiratory mucosa is more potent than serum IgG for cross-reactivity against diverse virus strains. IAV (H1N1 A/Yamagata/120/86) HA immunization can lead to protection of mice against infection with multiple virus strains [62,63]. In this case, while the protection rate was shown to be proportional to the level of cross-reactive Abs produced in the URT [63], the reduced level of the cross-reactive Abs evidently resulted in a decline in the efficiency of protection against heterologous virus infection [64]. It has been demonstrated that S-IgA purified from volunteers intranasally immunized with inactivated vaccine against the H5N1 has the potential to act as the broad neutralizing Ab against variant virus strains [60]. Unlike IgG, IgA does not promote the activation of the inflammatory complement pathway [65]. Thus, all the above responses are non-inflammatory in nature.

In response to antigen stimulation, IgA class switching and IgA secretion occur in B cells stimulated by specific signals such as co-stimulatory molecules and cytokines and in Th cells via T-cell-dependent and T-cell-independent pathways [66]. The above process is dependent on TGF-β signaling in B cells that activates isotype switching to IgA and is essential for T-cell-dependent and independent pathways. Notably, multiple activation signals contribute to the IgA isotype class switching. For example, CD40L binding to its receptor CD40 on B cells, binding of B-cell-activating factor or a proliferation-inducing ligand to transmembrane activator and calcium modulator and cyclophilin ligand interactor on B cells, IgA switching-directing TGF-βð1, and Th2-type cytokines facilitate IgA+ B cell post-switch and differentiation toward IgA-secreting plasma cells [67]. Furthermore, specialized T cells or DCs harboring mucosal inductive sites were found to be most effective for switching of sIgM+ sIgA- B cells to IgA-producing cells [68].

## 3. The Importance of Mucosal Immunity against Influenza Virus Infection

At present, the commercial vaccines against influenza available on the market mainly comprise inactivated influenza A virus (IIV) vaccine and recombinant influenza A virus (RIV) vaccine. Strains used in commercial influenza vaccines are selected based on the compositions of viruses from the surveillance, laboratory, and clinical observations [69]. These vaccines are licensed clinically only for intramuscular injection. The IIV vaccines mainly include whole inactivated virus (WIV), split virus, and subunit vaccines [70]. WIV vaccine production begins with the virus growth in 9–11-day-old pathogen-free embryonic chicken eggs, followed by chemical inactivation using formaldehyde or β-propiolactone, concentration, and purification. WIV has safe and complete antigenic components that are nontoxic and have strong immunogenicity and no risk of mutation. Although WIV vaccines usually provide protection against the virus subtypes that are antigenically closely related to one another, minor changes in antigenicity could cause a loss in the cross-reaction and protection [71]. In split virus vaccines, the virus envelope has been disrupted by treatments with diethyl ether or detergents for exposure of all viral proteins [72]. The subunit vaccines are prepared by extracting an immunogenic protein of influenza viruses and supplemented with a very safe adjuvant for stimulating production of sufficient immunity. The RIV vaccines are generated through a viral host (baculovirus Autographa californica nuclear polyhedrosis virus) in the insect cell line, which were authorized for use in the United States. These influenza vaccines, administered subcutaneously or intramuscularly, can elevate the level of serum Abs at systemic immune compartments, providing the most efficient, valuable, and low-cost means for effectively controlling the virus infection and subsequently reducing morbidity and mortality. However, all these parental administrations fail to elicit local mucosal immunity on the location of initial infection [73], representing a limited capacity of the conventional vaccines in conferring broad protective immunity against infections. Moreover, once the vaccine strains are not well matched to the seasonal circulating influenza strains, the effectiveness of these vaccines becomes very low.

The respiratory mucosa is the first target of influenza virus, constituting the first line of defense against the infection, which can effectively prevent the virus from entering [74]. Mucosal vaccines are designed to mimic the natural process of virus infections and evoke better mucosal immune responses than parenteral vaccines. On the one hand, while mucosal vaccines enhance innate immunity by increasing the expression of cytokines such as IL-4, IL-6, IL-10, and IL-21; chemokines; and co-stimulatory molecules, including CD40, CD80, CD86, IFNα, and B cell-activating factor, they induces differentiation of CD4+ T cells toward Th1, Th2, Treg, Th17, and Tfh cells. On the other hand, mucosal immunization can induce more S-IgA Abs than parenteral vaccines [75]. S-IgA Abs on the mucosal surface have the capacity to effectively prevent virus infection due to the fact that these Abs remain active on the surface prior to attachment of the pathogens to mucosal epithelium, the primary site for virus infection. Furthermore, S-IgA Abs display more effective protection against the influenza virus variants than serum IgG Abs because S-IgA Abs act more widely [76].

Moreover, it has been shown that mucosal vaccines induce cross-reacting Ab in the respiratory tract, providing cross-protection against heterologous virus infection. Inoculating the influenza HA vaccine together with cholera toxin B subunit (CTB) or B subunit of heat-labile enterotoxin (LTB) supplemented with cholera toxin (CT) intranasally enhanced anti-HA lgA and IgG Ab responses in nasal wash or serum, which protect mice against variant virus infection [77]. Okamoto et al. found that intranasal formalin-inactivated whole-virion H5N1 vaccine injection protected mice against challenges with lethal influenza viruses of homologous and heterologous subtypes by inducing cross-reactive neutralizing antibody responses and cross-reactive cell-mediated immune responses [78], suggesting the significant role of mucosal immunity in cross-protective efficacy. Besides, they also demonstrated that the cross-protection elicited by the whole-virion vaccine and the split-virion vaccine plus mucosal adjuvants is similar [79]. In addition, mucosal vaccination can avoid the need for needles, reducing accidents with syringes and causing less pain. Particularly, trained medical personnel are not always required to deliver nasal vaccines in the large-scale immunization program. Practically, exploration of mucosal immunization in AIVs defense could contribute to the development of effective vaccines and drugs against the viruses and identify the molecular pathogenesis related to influenza infection and the working mechanism underlying normal mucosal immunization.

## 4. Strategies for Mucosal Vaccination

Researchers have been attempting to create universal influenza vaccines for providing broad and sustainable protection against diverse influenza viruses. In recent years, research on the prevention of influenza infection by nasal immunity has gained more and more attention. To effectively work in mucosal immunization, mucosal vaccines must be designed to possess the characteristics of mucosa and promote the specific immune responses to vaccine antigens in the mucosa. Currently, generating effective mucosal vaccines mainly involves the identification of efficient means for antigen delivery to the mucosal immune system and the development of safe and effective mucosal adjuvants or immunoregulatory agents.

### 4.1. Live Attenuated Vaccines

A growing number of pre-clinical studies and clinical trials have advanced our understanding of how live attenuated influenza vaccines (LAIVs) elicit mucosal antibodies via intranasal administration. Contrary to the wild-type forms, attenuated viruses have the capacity to replicate well at temperatures around 25 °C rather than at 37 °C, deterring their replication in the lower airway, including lung tissue, and the subsequent occurrence of influenza-like disease [80]. The most important feature of LAIVs is that they elicit a minor infection at the site of challenge and present a high degree of antigen exposure [81]. Ideally, LAIVs have been constructed to possess a limited replication capacity to avoid undesirable inflammatory response while delivering a sufficiently high dose of antigens at the site of administration [82].

Among developing live attenuated mucosal vaccines, the major class of vaccines, oral vaccines, are currently being assessed in clinical studies. Oral vaccines are classified into intranasal and sublingual vaccines that are developed for the control of influenza, rotavirus, norovirus and measles viruses [83]. Experimental LAIVs have been found to be effective in poultry [84]. LAIVs have been administered to adults in Russia since the 1950s. Starting from 1987, LAIV administration in Russia has been implemented throughout various age groups [85,86]. LAIVs were licensed in the US in 2003 for use in healthy individuals aged 2–49 years, while in the EU in 2012 for normal children aged 2–17 years [87].

Successful development of LAIVs largely rests on the proper balance between attenuation and immunogenicity. It has been reported that serial virus passage in the host cells during vaccine development causes a decrease in the virulence of effective live attenuated vaccines for rotavirus [88]. As a result, the above vaccines are sufficiently attenuated, eliciting a minor subclinical infection, while retaining a high immunogenicity. In the other case, the live oral typhoid vaccine exhibits moderate immunogenicity but good safety due to extensive attenuation [89]. Live attenuated H5N1 influenza viruses were produced by removing polybasic cleavage site of HA protein and selected gene deletion, leading to generation of a safe vaccine with high immunogenicity [90]. Therefore, precisely targeted gene deletion provides a controlled approach for attenuation that can be highly desirable to generate a safe and stable live vaccine. Further studies on the genetic basis of attenuation could make a great contribution to development of the next generation of live vaccines and improvements in the safety and stability of the vaccines.

### 4.2. Mucosal Adjuvants

Adjuvants can potentially increase the immunogenicity of mucosal influenza vaccines, while enhancing the efficacy of vaccines through modulating the quantity and quality of host immune responses. Commonly used adjuvants include a wide range of compounds (Table 1). The commercial mucosal adjuvant in food animals is rare. Numerous experimental animal studies have demonstrated that enterotoxins (cholera toxin and heat-labile enterotoxin), TLR ligands (Poly I:C, lipopolysaccharide, flagellin, CpG), chicken interleukin-1 beta, etc. elicit increased immune responses and protect against viral challenge in poultry and other animals (Table 1). These should be good candidates for mucosal influenza vaccines for clinical use in poultry or other food animals in the near future. For most inactivated vaccines, multiple administration is needed to attain protective levels in recipients; co-administration with an adjuvant could promote the immune response to the initial dose of vaccines, resulting in an elevated response above the threshold of protection. Importantly, formulation of vaccines with adjuvants not only require a lower amount of vaccine agents and less number of doses for inducing protective immune responses, but increase the efficacy of vaccines as well. Compared to the standard response induced by 15 mg H5N1 antigen, administration of AS03 elicited a decreased response by 3.75 mg [91]. Mucosal vaccination could offer an advantage in inducing local immunity at the site of infection to systemic vaccination. However, mucosal surfaces display a broad tolerogenicity and harbor a number of antigen barriers, such as cilia (mechanical), mucus (chemical), and proteolytic enzymes (biochemical). As a result, mucosal vaccination could be a challenge. To address the above issues, special adjuvants may be used in mucosal vaccination for antigen protection and induction of the local immunity [92].

Adjuvants act via the following mechanisms: (1) a depot formation at the site of injection, which enables the vaccine agents to be slowly released and the host immunity to be constantly stimulated; (2) enhancement of the agent uptake via APCs and APC activation and maturation; (3) immune cell recruitment; and (4) innate immune receptor activation. Moreover, adjuvants can shape subsequent adaptive immunity for producing the most effective and protective responses to a given pathogen through targeting the subsets of innate immune cells and forming a unique cytokine milieu associated with Th1, Th2, and/or Th17.

A number of adjuvants for inactivated influenza vaccines including TLR agonists are widely used to enhance the vaccine immunogenicity (Table 1). Studies on innate immune system have identified PAMPs as adjuvants used in animals such as mice, non-human primates, and chicken. For example, poly(I:C) and CpG, the respective ligands of TLR3 and TLR21, stimulate elevated levels of S-IgA in the airway and IgG against AIVs in the serum when given along with inactivated H5N1 [93]. It has been shown that intranasal administration of formalin-inactivated influenza H5N2 vaccines formulated with Salmonella flagellin enhanced the nasal IgA production in chickens [94]. In addition, CpG ODN has the potential to induce a robust IFN-γ response that acts in APC maturation and promotes antigen presenting abilities of APCs [95] while facilitating B cell activation and subsequent expression of co-stimulatory molecules MHC class II and cytokines [96]. This characteristic allows CpG ODN to be widely used in chickens for increasing the immunogenicity of vaccines against mucosal pathogens, including AIVs.

### 4.3. Mucosal DNA Vaccines

DNA vaccines are capable of expressing immunogenic antigens in vivo, thereby inducing cellular and humoral immunities without causing host exposure to live viruses [116]. Extracellular proteins can be selected as the DNA vaccine antigens that are recognized by MHC class I or II directed intracellular proteins. Notably, DNA vaccines have potential advantages over conventional vaccines, including ease of manufacture, stability at room temperatures, and the ability to mimic natural infections and elicit an appropriate immune response. Thus, DNA vaccines without the need for virus propagation in eggs represent a fast, safe, and efficient alternative to conventional influenza vaccines [117]. In the past two years, testing of DNA vaccination for targeting the mucosa has revealed promising results. DNA vaccination via mucosal (oral, intranasal, and vaginal) routes is capable of generating mucosal and systemic immunities.

It has been shown that intranasal administration of DNAs in mice leads to a fast and relatively even distribution of plasmid DNAs all over the body [118]. DNA vaccines are delivered as naked plasmid DNA, resulting in a weak immunogenicity due to nuclease-mediated DNA degradation and inefficient delivery to the target cells. To optimize DNA vaccination, various strategies involving adjuvant formulation, distinct prime-boost combinations, and diverse approaches for delivery have been proposed to enhance the immunogenicity of DNA vaccines. Compared with the DNA vaccine alone, a-Galactosylceramide-adjuvanted DNA vaccine can induce a marked increase in titers of serum IgG and IFN-γ levels in mice [119]. Other approaches have been used to increase vaccine immunogenicity by targeting the DNAs to M cells (specialized epithelial cells in the mucosa). One study showed that nasal delivery of complexes formed from DNA and M cell-binding σ1 protein in reovirus produced strong antigen-specific responses with mucosal IgA and IgG, particularly CTL-stimulated responses to DNA-encoded antigen in the lung, indicative of accumulation of Th1 and Th2 effector cells in the mucous tissues [120].

### 4.4. Mucosal Delivery Systems

#### 4.4.1. Particulate Formulations

Particulate vaccines are capable of forming a close contact with the epithelial cells in the mucosa via included anchoring devices with adhesive properties (lectins, specific Abs) or included immunomodulating factors (TLR ligands, TLR ligand binding receptors) [83,121]. The above formulation involves virus-like particles (VLPs), biodegradable microparticles, and nanoparticles including lactide-co-glycolide-based microparticles. VLPs are generated by viral structural proteins alone without any genomic composition. These non-replicating particles are characteristic of retaining virus morphology and antigenicity. Therefore, they are capable of activating innate immune responses. Oral administration of influenza VLPs was found to induce serum IgG and S-IgA responses [122]. VLPs were identified to be promising for “universal” vaccines with broad cross-protection, and triple-subtype vaccine (H5, H7, and H9) had the capacity to protect chickens against heterologous virus challenge including HPAI H5N2, HPAI H7N3, and LPAI H9N2 [123]. Nanoparticle-based vaccines have the potential advantage in overcoming the low immunogenicity and inefficient delivery, while maintaining slow antigen release and promoting antigen presentation for desired immune responses. Multiple studies reported that NLRP3-associated inflammasome can be activated by nanoparticles such as carbon black, poly-lactic-co-glycolic acid (PLGA) and polystyrene, TiO2, SiO2, and aluminum oxyhydroxide [124]. For instance, PLGA nanospheres were proved to be good candidates for AIV defenses, as PLGA-based vaccines induced serum IgG and S-IgA as well as a mixed Th1/Th2 response [125,126].

#### 4.4.2. Live Vector Vaccines

Recombinant technology has been employed to engineer live vector vaccines to express target antigens. The vectors usually have minimal replicative ability to allow immune stimulation without causing infection. Delivery of vector vaccines via the mucosal routes mimics the natural infection, inducing strong mucosal, systemic, and cellular immunities against micro-organisms. Many candidate vectors. e.g., bacillus subtilis [127], Fowlpox virus [128], herpesvirus of turkey (HVT) [129], Newcastle disease virus (NDV) [130] were used for AIVs defenses. For mucosal vaccination, live vector vaccines can simultaneously express different antigens to prevent and treat a variety of diseases. The antigen presenting system can be targeted to the mucosa in which it survives and multiplies, and then carried antigens of the vaccines are expressed as the bacteria or viruses proliferate in the body. As one of the most characterized viral vectors, NDV has been utilized for expressing diverse influenza antigens, including HA and NA [130,131]. Studies on H5N1 or H7N9 HA expressing NDV have obtained positive results [130,132]. H5 and H7 vaccines using NDV as the vector can elicit high levels of Abs against HI and protect chickens from infection with H7N9 or HPAI H5N1 virus, indicative of the effectiveness of NDV-vectored influenza vaccines. Likewise, HVT has been used as a viral vector for constructing avian influenza vaccines. A recombinant vector vaccine rHVT-H9 expressing the H9N2 HA was shown to be capable of inducing robust cellular and humoral immunities in chickens. Strikingly, it was found that rHVT-H9 causes no severe adverse effects, showing its potential as a universal influenza vaccine [129].

### 4.5. Mucosal Vaccine Delivery Routes

#### 4.5.1. Oral Vaccination

Administration of mucosal vaccines via the oral route is strongly recommended because it avoids an injection, is safe and painless, and does not require professionals for delivery. However, the efficacy of oral vaccination has proven less satisfactory due to the fact that vaccine degradation occurs in the harsh environment of the gastrointestinal tract, and mucosal tolerance suppresses immune responses to a foreign antigen. Thus, oral vaccination may require highly sophisticated formulations involving approaches for cell-specific targeting and immunomodulation. In the mucosal tissues, epithelial cells are coated by a viscous fluid called mucus containing a layer of glycoproteins (mucins). The layer of mucus serves as a physical barrier between the epithelium and invaded pathogens while harboring competitive binding sites for entrapping microbes and S-IgA Abs for binding to and neutralizing pathogens. Structurally, the intestinal epithelium formed by a single-cell layer constitutes the largest and most important barrier to the entry of foreign substances and functions as a selective barrier that only allows for nutrients and electrolytes to be absorbed while preventing the entry of intra-luminal antigens, toxins, and microorganisms [133,134]. The selectivity of the epithelium relies on establishment of tight junctions that serve as the rate-limiting barrier of intestinal permeability. The mucosal epithelial cells can secrete antigen-degrading enzymes. Indigenous microorganisms colonizing a mucosal surface form a solid foothold for the mucosal epithelium or the mucus, thereby preventing a direct interaction between the antigens or their delivery systems and the epithelium. Given that oral vaccines are subject to a dilution prior to being absorbed in the mucosa, a degradation by proteases or nucleases present in the stomach and mucus or an exclusion by tight-junction-based barrier in the epithelium, oral vaccination requires a relatively larger amount of vaccines for eliciting effective immunity against the pathogens. However, precise quantitation of the vaccine dose required for crossing the mucosa remains a significant challenge. Generally, repeated administration of higher doses of oral mucosal vaccines or design of the vaccines mimicking the structure of immunogenic microbes may achieve an effective immunization [134].

There exist continued efforts in developing oral live attenuated vaccines. However, compared with live-attenuated pathogen based vaccines with limitations in safety, recombinant subunit vaccines or non-living or non-infectious vector vaccines could be more attractive, albeit more difficult to deliver [134,135]. Hence, it is important to develop live mucosal vaccines with stronger immunogenicity, better stability, and increased safety. Although there are several hurdles in achieving the above goal, effective oral vaccines such as rotavirus and cholera vaccines have been generated. However, it remains to be understood why only a proportion of oral vaccines work. Thus, it would be impossible to successfully generate effective oral vaccines by simply replicating the formulations or protocols. Nevertheless, the effective oral vaccines may be attributed to the delivery of sufficient antigens within sustained time periods for effectively initiating intestinal immune system via the follicular epithelium and Peyer’s patches.

#### 4.5.2. Intranasal Vaccination

As a major entering portal for pathogens, the nasal route becomes a promising option for mucosal vaccination due to its unique physiological and immunological characteristics. Intranasal vaccination is capable of stimulating immune responses in the nasopharynx-associated lymphoid tissue, eliciting systemic and mucosal immunities in the gastric mucosa and the respiratory and/or genital tract [136]. Most soluble antigens are not efficiently taken up and transported in the nasal cavity due to its anatomical and physiological features. Generally, subunit antigens with very little affinity for the nasal epithelium display a poor immunogenicity and are quickly eliminated via mucociliary clearance. The tight junctions of nasal epithelial cells can remarkably decrease the epithelial permeability to macromolecules [137]. Moreover, nasal enzymes and local pH are among the factors affecting the stability of soluble antigens. Overall, the formulation of nasally administered vaccines needs to be optimized for a prolonged residence time in the nasal mucosa and increased stability [134]. However, intranasal vaccination could be superior to oral vaccination as the former requires markedly lower doses of antigen and/or adjuvant. It has been proved that intranasal administration of LAIVs can effectively protect from the seasonal infection and even induces cross-protective immune response against drifted strains [138]. Novel approaches for the co-delivery formulation have been employed to carry out one or more of the following functions: mucosal adhesiveness, protection, permeation and enhanced penetration of antigens, inductive-site specific targeting, and adjuvant effect.

## 5. Protective Mucosal Immunity to Newcastle Disease in Chickens: A Successful Case for Vaccine Design

Vaccination via the nasal cavity is the earliest mean of mucosal delivery, and nasal immunity can effectively prevent respiratory diseases. At present, mucosal immunity has made great contributions to human medicine from theory to practice, and various influenza vaccines such as LAIVs and cold-adapted influenza attenuated vaccines have been developed [139,140]. The oculo-nasal-oral route of vaccination is likely to be the most practical approach for preventing infectious diseases in poultry. Immunization of Newcastle diseases has demonstrated that mucosal immunization is of good efficacy. Currently, nasal and eye-based immunization has been successfully applied to the large-scale prevention of Newcastle diseases in chickens. The nasal and eye-based routes were used to deliver Newcastle disease attenuated vaccines. These delivery routes produce S-IgA Abs in the airway, digestive tract, and Harder gland and induce high titers of serum IgM and IgG. This suggests that mucosal immunity may contribute to establishment of a good defense line, which can effectively control the spread of the viruses. It was found that serum IgA and IgM against NDV appear within the first week following vaccination, whereas IgG can be detected starting from the second week. In this case, induction of cellular immunity against NDV occurred in the first week following vaccination [141]. In brief, the case of Newcastle disease control demonstrates that mucosal immunization is a rapid, efficient, and economical method to prevent respiratory diseases.

## 6. Conclusions and Perspectives

Avian influenza is a serious respiratory disease. Although currently used inactivated vaccines are effective, circulation of antigenically different strains could reduce the efficacy of vaccines due to the fact that almost all vaccines are administered via the i.m. route. The rate of antigenic variation of AIVs is accelerating, thereby increasing the risk of human infection and harm to the economy and society. Therefore, speeding up the research on AIVs vaccines and improving vaccine safety and reliability will have huge economic and social benefits. Mucosal immunity constitutes the first line of defense against AIVs; the mucosal delivery route elicits mucosal and systemic immunities simultaneously. Compared to inactivated vaccines, mucosal vaccines stimulate both cellular and humoral immunities and can be more effective in protecting against influenza variant strains, hence producing more cross-reactive and longer-lasting comprehensive immune responses. Overall, mucosal vaccines and delivery routes should play an irreplaceable role in remarkably reducing AIVs-caused burden.

## Figures and Tables

**Table 1:** Representative nasally or orally administered vaccine adjuvants and immune responses.

Type of Adjuvant	Composition	Target	Delivery Route	Immune Responses
Enterotoxins	Cholera toxin	GM1	Nasal or oral	Increased specific IgA and IgG [97]
	the subunit B of cholera toxin	GM1	Nasal or oral	Increased specific IgA, IgG and T cell response [98,99]
	Mutant Escherichia coli heat-labile enterotoxin	GM1 and other gangliosides	Nasal or oral	Increased S-IgA, IgG and T cell response, enhanced IFN-γ, IL-6 and IL-10 cytokine secretion [100,101]
TLR ligands	Poly I:C	TLR3	Nasal	Decreased oropharyngeal and cloacal virus shedding [93,102]
	Lipopolysaccharide	TLR4	Nasal or oral	Decreased oropharyngeal and cloacal virus shedding [102,103]
	Flagellin	TLR5	Nasal or oral	Increased IgY and IgA, protection of lethal viral challenge [94,104]
	CpG	TLR9/TL21	Nasal or oral	Increased S-IgA, IgG and IFN-γ [105,106,107,108]
	Variant-specific surface proteins	TLR4	Oral	Increased IgA, IgG and IFN-γ, protection of viral challenge [109]
Mucoadhesives	Chitosan	Tight junctions	Nasal or oral	Increased IgG and IFN-γ, 100% protection for fowl typhoid [110,111]
	Lectins	M cells	Nasal	IgG and IgA induction, induced heterosubtypic immunity [112]
Cytokines	chicken interleukin-1 beta	IL-1R	Nasal	Increased specific IgA, S-IgA and IFN-γ [113]
	IFN-λ	M cells	Nasal	IgG1 and IgA induction, protection of viral challenge [114]
Synthetic adjuvant	SF-10	Dendritic cells	Oral	Increased specific IgA, S-IgA, IgG and cytokine production [115]
